# The dual role of the glycosaminoglycan chondroitin‐6‐sulfate in the development, progression and metastasis of cancer

**DOI:** 10.1111/febs.14748

**Published:** 2019-02-05

**Authors:** Adam Pudełko, Grzegorz Wisowski, Krystyna Olczyk, Ewa Maria Koźma

**Affiliations:** ^1^ Department of Clinical Chemistry and Laboratory Diagnostics School of Pharmacy with the Division of Laboratory Medicine in Sosnowiec Medical University of Silesia Katowice Poland

**Keywords:** chondroitin‐6‐sulfate, cancer, extracellular matrix

## Abstract

The remarkable structural heterogeneity of chondroitin sulfate (CS) and dermatan sulfate (DS) generates biological information that can be unique to each of these glycosaminoglycans (GAGs), and changes in their composition are translated into alterations in the binding profiles of these molecules. CS/DS can bind to various cytokines and growth factors, cell surface receptors, adhesion molecules, enzymes and fibrillar glycoproteins of the extracellular matrix, thereby influencing both cell behavior and the biomechanical and biochemical properties of the matrix. In this review, we summarize the current knowledge concerning CS/DS metabolism in the human cancer stroma. The remodeling of the GAG profile in the tumor niche is manifested as a substantial increase in the CS content and a gradual decrease in the proportion between DS and CS. Furthermore, the composition of CS and DS is also affected, which results in a substantial increase in the 6‐*O*‐sulfated and/or unsulfated disaccharide content, which is concomitant with a decrease in the 4‐*O*‐sulfation level. Here, we discuss the possible impact of alterations in the CS/DS sulfation pattern on the binding capacity and specificity of these GAGs. Moreover, we propose potential consequences of the stromal accumulation of chondroitin‐6‐sulfate for the progression and metastasis of cancer.

## Graphical abstract

The remarkable structural heterogeneity of chondroitin sulfate (CS) and dermatan sulfate (DS) and the huge binding potential of these glycosaminoglycans make them important regulators of biological processes. The remodeling of CS/DS associated with tumor development manifests as a progressive accumulation of chondroitin‐6‐sulfate in the extracellular matrix. The possible impact of this glycan on the progression and metastasis of cancer is discussed.

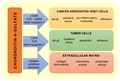

**Abbreviations**
C‐4‐S: chondroitin‐4‐sulfate
C‐6‐S: chondroitin‐6‐sulfate
CNS: central nervous system
CS: chondroitin sulfate
CST: chondroitin sulfotransferase
DS: dermatan sulfate
ECM: extracellular matrix
FGF: fibroblast growth factor
GAG: glycosaminoglycan
GalNAc: *N*‐acetyl galactosamine
GlcA: glucoronate
HA: hyaluronan
HARE: HA receptor for endocytosis
HGF: hepatocyte growth factor
Hyal: hyaluronoglucosaminidase
ICAM: intercellular adhesion molecule
IdoA: iduronate
IL: interleukin
LPS: lipopolysaccharide
MDK: midkine
MMP: matrix metalloproteinase
NF: nuclear factor
NSCLC: non‐small cell lung cancer
PDGF: platelet‐derived growth factor
PG: proteoglycan
PTN: pleiotrophin
RAGE: receptor for advanced glycation endproducts
sCD44(: standard CD44)
TGF: transforming growth factor
TIMP: tissue inhibitor of metalloproteinases
TLR: Toll‐like receptor
UA: hexuronate
VEGF: vascular endothelial growth factor

## Introduction

The local microenvironment of living cells, referred to as the tissue stroma or niche, consists of host cells and an extracellular matrix (ECM) that surrounds them. The ECM is a conglomerate of molecules that interact with one another, and is mainly composed of collagen, fibronectin and elastin, which form the matrix fibrillar network, and by hyaluronan (HA), tenascins and glycosaminoglycan (GAG)‐containing glycoproteins 1. The latter, called proteoglycans (PGs), can be associated with the cellular membrane as components of its glycocalyx or they can form matrix macrocomplexes. The variable content and arrangement of the ECM molecules determines the distinctive biochemical and biomechanical properties of the stroma of a given tissue 2. However, the ECM is not just a static fibro‐hydrated scaffold that is modified in response to growth or repair 3. It is now clear that the ECM microscopic topology is continuously being remodeled 3. This matrix remodeling is precisely regulated since it is the result of intracellular synthesis, post‐translational modifications, secretion, and finally the extracellular degradation of the ECM components 3. This sequence of events determines a tissue‐dependent turnover time for a given molecule and/or macrocomplex under local stroma conditions. Moreover, enzyme‐dependent ECM processing can release fragments of the matrix components and/or sequestrated molecules, which have a significant biological activity 2. Thus, ECM remodeling generates specific ligand patterns that can be precisely organized in a regulated manner, accessibility and direction toward appropriate cell receptors 3, 4, 5. These signals are capable of significantly influencing cell behavior 6. In this way, the ECM is capable of modulating various cell functions that range from cell proliferation, adhesion and migration to cell differentiation and cell death 5. Interestingly, disturbances in ECM remodeling can promote or sometimes initiate tumor growth 2, 7. Moreover, tumor cells are able to manipulate the local stroma conditions in order to enhance their own survival, thereby creating a positive tumorigenic loop 3. Once tumor‐supporting microenvironmental conditions are created, they play a prominent role in the growth, progression and spread of cancer 8. On the other hand, it has been found that the ECM of the early stage embryonic mesenchyme from the mouse mammary gland is sufficient to suppress the growth and to induce the differentiation of mammary cancer cells both *in vitro* and *in vivo* 9. Interestingly, the mesenchymal deposition of biglycan, which is PG substituted with two dermatan sulfate chains, is required to exert this biological effect 9. Biglycan and other commonly spread matrix PGs that are substituted with chondroitin sulfate (CS) or dermatan sulfate (DS), such as decorin or versican, affect cellular function both directly and indirectly through an impact on the formation of the ECM architecture as well as through binding to and influencing the activity of various functional molecules (growth factors, cytokines, cell‐surface receptors or enzymes) 10, 11. However, we should bear in mind that these PGs, especially decorin, can exert antagonistic effects that can either promote or inhibit the progression of a tumor depending on its origin 12.

## CS and DS are both structurally heterogeneous

**Figure 1:**
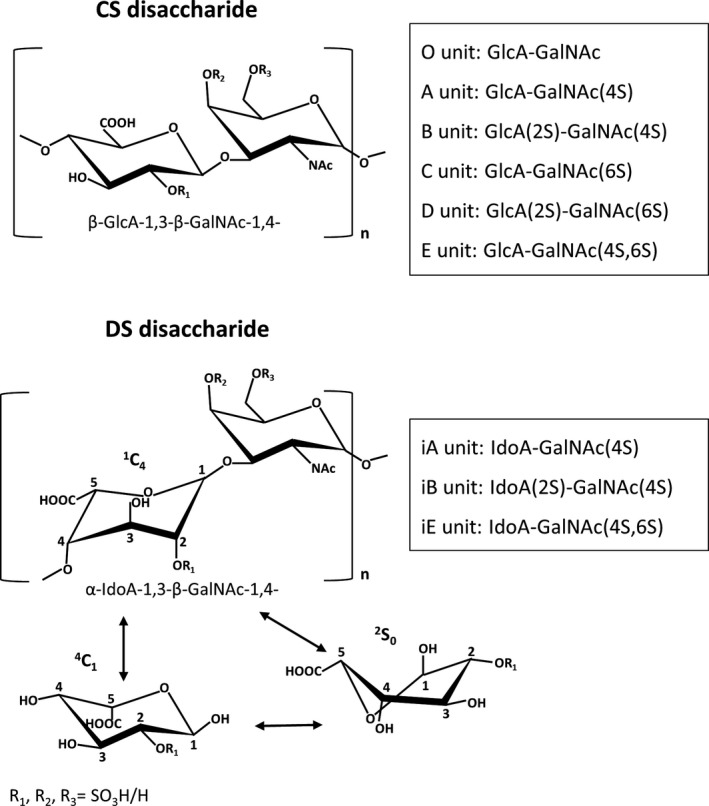
The structure of disaccharide units in chondroitin sulfate/dermatan sulfate (CS/DS) chains as well as conformations of iduronate residue. GalNAc, *N*‐acetylgalactosamine residue; GlcA, glucuronate residue; IdoA, iduronate residue; R, possible position of esterification by sulfate.

The complex role of PGs in the biology of a tumor is at least in part defined by tissue localization of these molecules, their local content and their fine structure 13, 14, 15. The last feature is especially related to their GAG portion. CS and DS, which are attached to the majority of the ECM‐localized PGs, contain an *N*‐acetylgalactosamine (GalNAc) residue in their disaccharide monomers. In addition to this hexosamine, the CS disaccharide unit is composed of a glucoronate (GlcA) residue, whereas a typical DS disaccharide unit contains IdoA residue (Fig. 1). However, DS chains are commonly copolymers of IdoA‐ and GlcA‐containing disaccharides 11. In contrast to GlcA, which assumes only a ^4^C_1_ chair conformation, IdoA displays a greater conformational flexibility as it can adopt one of three different spatial conformations (i.e. the ^1^C_4_ and ^4^C_1_ chairs and the ^2^S_0_ skew‐boat) 16 (Fig. 1). Thus, the contribution of IdoA into DS facilitates the spatial fitting of this GAG chain to bound ligand 16, 17. The biosynthesis of CS/DS relies on the alternating incorporation of the GalNAc and GlcA residues into the non‐reducing end of a growing carbohydrate chain and is dependent on the orchestrated action of several enzymes 18, 19. A newly formed chondroitin chain, being a common precursor of CS/DS, which is covalently bound to core protein via a tetrasaccharide linkage region, is further subjected to enzymatic modifications such as the sulfation and/or epimerization of its monosaccharide residues. The epimerization of GlcA residues at the C5 position into IdoA residues refers exclusively to the DS chains and is catalyzed by one of two glucuronosyl 5′‐epimerases 20. The final percentage contribution of IdoA to DS composition is determined by the subsequent sulfation at the C4 of the adjacent GalNAc residues 21. This 4‐*O*‐sulfation prevents the reversion of the epimerization and increases the extent of this process 22. Both the number and the localization of the IdoA and GlcA residues in the DS chains are variables that are reflected in a tissue‐specific glucuronosyl epimerization pattern of this GAG 23, 24. The biological relevance of the epimerization pattern can be deduced by the results, which show a linear correlation between the decreasing iduronate content in decorin and biglycan and bone aging 25. Moreover, recent findings have suggested that even a trace level of IdoA in the DS chains of decorin and biglycan may be responsible for a milder phenotype of Ehlers–Danlos syndrome 26, 27. In contrast to glucuronosyl epimerization, the sulfation of some monosaccharide residues concerns both CS and DS, and is catalyzed by the appropriate *O*‐sulfotransferases 18. This modification most frequently affects the GalNAc residues, which are substituted by a sulfate group at the C4 and/or C6 position (Fig. 1). The 6‐*O*‐sulfated GalNAc residues predominate in chondroitin‐6‐sulfate (C‐6‐S), whereas chondroitin‐4‐sulfate (C‐4‐S) has a higher level of the 4‐*O*‐sulfated ones 19. Moreover, a huge majority of the GalNAc residues also undergo the 4‐*O*‐sulfation in DS 19, 20. The small amount of 6‐*O*‐sulfated GalNAc that is detected in this GAG most probably originates from the GlcA‐containing disaccharides since the presence of the IdoA‐GalNAc6S units, although suggested 28, is clearly not proved 20. In turn, some IdoA residues are modified by the sulfate group at the C2, while this occurs less frequently in the GlcA residues 18 (Fig. 1). Moreover, some of the disaccharides in CS/DS can be modified by more than one sulfate group, thereby creating four oversulfated analogs: UA2S‐GalNAc4S (where UA is a hexuronate residue) (B unit), GlcA2S‐GalNAc6S (D unit), UA‐GalNAc4S6S (E unit) and ΔUA2S‐GalNAc4S6S (Tris) (Fig. 1). The contribution of the oversulfated disaccharides to the structure of the CS/DS chains is believed to have an important impact on their binding to various biological molecules (for review: 29). The spatiotemporally specific regulation of the activity of sulfotransferases gives rise to the structural diversity of CS/DS, thereby resulting in a differential number and localization of the sulfate groups within the carbohydrate backbone, which is reflected in the tissue‐ and temporally specific sulfation pattern of these GAGs (for review: 30). In addition to this diversity in the degree of modification, the CS/DS chains are also composed of a variable number of disaccharide units, which is reflected in the differential molecular mass of these chains. CS and DS chains have an average distribution of 5–50 and 15–40 kDa, respectively 31. Thus, all of the above‐mentioned structural characteristics of CS/DS are responsible for the remarkable structural heterogeneity of these GAGs 32. As a result, CS/DS are predicted to contain structural microdomains endowed with specific biological information that is translated into specific binding abilities 29. CS/DS binds to ligands such as cytokines and growth factors including fibroblast growth factor (FGF)‐2, FGF‐7, FGF‐10, FGF‐18, hepatocyte growth factor (HGF), midkine (MDK), platelet‐derived growth factor (PDGF), pleiotrophin (PTN), vascular endothelial growth factor (VEGF), transforming growth factor (TGF) β, human β‐defensin 2 and/or various cell surface receptors such as contactin‐1, receptor for advanced glycation endproducts (RAGE), cMet receptor for HGF, FGF receptors, selectin P and L, intercellular adhesion molecule (ICAM)‐1 and CD44, as well as extracellular enzymes 33, 34, 35, 36, 37, 38, 39, 40, 41, 42, 43, 44, 45, 46, 47. Based on these interactions CS/DS can substantially affect the behavior of cells.

## The metabolism of CS/DS is strongly altered in the tumor stroma

Tumor‐associated changes in the stroma of human malignant lesions are reflected in an increased content of chondroitin‐originated GAGs. This accumulation is accompanied by an enhanced expression of *CHSY1*, encoding chondroitin synthase 1 48, 49. However, it is possible that the enzyme is also responsible for the appearance of special structures in CS/DS that promote the progression of a tumor as hypothesized in the case of hepatocellular carcinoma 48. The remodeling of the GAG profile in the majority of, if not in all, tumors is manifested as a substantially increased content of CS in the tissues 50, 51, 52, 53, 54, 55, 56, 57, 58, 59, 60, 61, 62, 63, 64, 65, 66, 67, 68, 69. In turn, the level of DS in a tissue depends on the type of tumor and is mostly reduced. However, in some cancers such as liver, lung, pancreatic, colorectal and gastric cancers as well as in the breast fibroadenoma, the stromal content of DS is elevated 53, 54, 55, 56, 59, 61, 64, 70. Nevertheless, in light of the significant CS accumulation, the CS/DS remodeling in the stroma of all of the human epithelial tumors can be considered to be a DS to CS shift. This shift in the microenvironment of tumors may partly result from alterations in the PG profile of a tissue 50, 51, 60 since an abnormal expression of the PG core proteins can lead to changes in the type and structure of the GAG chains that are attached 71. However, tumor‐associated CS/DS remodeling can also be the result of disturbances in the GAG‐synthesizing and/or modifying machinery. These disturbances are manifested as a changed sulfation pattern of CS/DS that has common features regardless of the tumor type. First and foremost, a substantial increase in the content of the 6‐*O*‐sulfated and/or unsulfated disaccharides that is observed in a huge majority of cancers is concomitant with a reduction in the 4‐*O*‐sulfation level 55, 59, 60, 62, 72, 73, 74, 75, 76, 77, 78, 79, 80, 81, 82 (Table 1). The observed loss of 4‐*O*‐sulfated GalNAc may be one reason for the disappearance of the IdoA residues despite the tumor‐associated upregulation of glucuronic epimerase, which has been found to be characteristic in human cancers such as squamous carcinoma or glioblastoma 83, 84. The tumor‐associated remodeling of the CS/DS sulfation pattern can also affect the disulfated disaccharide content. Unfortunately, only a few studies have focused on this aspect of CS/DS metabolism in the tumor niche 72, 74, 75, 78, 81, 85, 86. These investigations have reported that the contribution of various disulfated disaccharides to the composition of CS/DS is cancer type‐ and cancer grade‐dependent 72, 74, 75, 78, 81, 85, 86 (Table 1). The accumulation of disulfated disaccharides in the CS/DS that exist in the tumor microenvironment can substantially modulate the progression and metastasis of a tumor due to the increased binding potential of these disaccharides 87, 88. The significance of CS sulfation on growth factor‐mediated signaling as well as for the progression and metastasis of cancer is now being exhaustively investigated (for review: 89). In addition to the tumor‐associated remodeling of the sulfation and/or epimerization patterns of CS/DS, alterations in the molecular mass distribution of these GAG chains have also been detected. This phenomenon, which is manifested as a shortening or lengthening of the CS/DS chains in comparison to their size in a normal tissue, seems to be dependent on both the type and the grade of a tumor 59, 62, 72, 73, 90, 91. The remodeling of the size of CS/DS in the tumor microenvironment can result not only from disturbances in the GAG‐synthesizing machinery but also from alterations in the extracellular processing of these molecules. The last process is primarily mediated by several members of the hyaluronidase family such as hyaluronoglucosaminidase (Hyal) 1 and 4 as well as sperm adhesion molecule 1 92. It seems that the former enzyme makes the greatest contribution to the catabolism of CS/DS in the extracellular space 93. Changes in the Hyal1 expression in the tumor microenvironment are dependent on the type of a tumor and correlate with its malignancy grade 94, 95. However, it is unknown whether this correlation results from Hyal1 impact not only on HA hydrolysis 94, 95 but also on CS/DS degradation in the tumor niche.

**Table 1:**
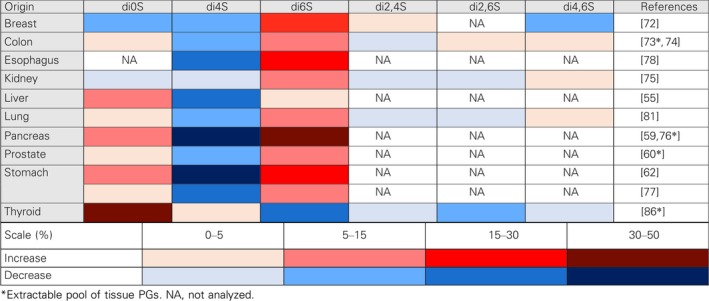
The remodeling of the sulfation pattern of chondroitin sulfate/dermatan sulfate in the human cancer stroma compared to healthy tissue. Results are expressed as changes in the percentage content of individual disaccharides

For several years, the remodeling of the CS/DS profile that is observed in the tumor niche has been considered to be required for the formation of more permissive conditions for the development, progression and metastasis of a cancer. It has been hypothesized that the elimination of DS from the tumor stroma may be due to its inhibitory effect on cancer growth and invasiveness. Indeed, DS has been found to inhibit the proliferation of some osteosarcoma and melanoma cell lines 39, 96. Moreover, DS‐bearing decorin was 20‐fold more effective than CS‐bearing decorin in inhibiting the mobility of osteosarcoma cells 97. However, DS can also stimulate the migration of cancer cells via promotion of HGF activity 78. On the other hand, numerous studies have shown a positive concentration‐dependent influence of CS on the growth and progression of tumor cells 98, 99, 100. The CS level also correlates positively with the more aggressive forms of cancer that are reflected in a higher histological grade or in a poorer patient prognosis 48, 49, 85. However, the final effect of CS/DS on cancer cells may be both tumor‐specific and depend on the concentration and structure of the GAGs 36, 85, 101, 102. Moreover, recent findings have suggested that the tumor niche‐associated CS/DS remodeling, especially a high deposition of C‐6‐S in the microenvironment of a tumor, may have more complex consequences.

## Insights into the biological relevance of 6‐*O*‐sulfation of CS/DS

The 6‐*O*‐sulfation of the GalNAc residues in the CS/DS chains is catalyzed by two chondroitin sulfotransferases (CSTs) – C6ST‐1 (encoded by the *CHST3* gene) and C6ST‐2 (encoded by the *CHST7* gene), which only overlap partly with respect to their expression pattern and substrate specificity 19, 103. Of these two enzymes the former, due to its prevalence in tissues, is considered to be responsible for the modification of CS and probably DS to the highest degree 19, 103. C6ST‐1 also controls the level of the 2,6‐*O*‐sulfated disaccharides in these GAGs since the 2‐*O*‐sulfation of uronate is preceded by the 6‐*O*‐sulfation of GalNAc 19, 104. The missense mutations that have been found in the gene of human C6ST‐1 are manifested clinically primarily in the development and maintenance of the skeleton, with a phenotypically broad spectrum of disturbances 105, 106, 107, 108. By contrast, a knockout of mouse C6ST‐1 has limited biological consequences that include a reduction in the number of splenic naïve leukocytes and an impaired regeneration of the nigrostratial TH‐positive axons despite the almost complete elimination of the 6‐*O*‐sulfation in CS/DS 109, 110. Thus, the different biological consequences of a C6ST‐1 deficiency in humans and mice suggest that differences in the biological importance of 6‐*O*‐sulfation in CS/DS may exist between species. On the other hand, the progressive accumulation of CS with enhanced 6‐*O*‐sulfation is not only distinctive for the tissue remodeling that is associated with the progression of a tumor, but is also observed during several physiological and pathological events. The significant diversity in proportions between the 6‐*O*‐ and 4‐*O*‐sulfated CS disaccharides is associated with the development and aging of the central nervous system (CNS). The increased level of 6‐*O*‐sulfated CS disaccharides that occurs in embryonic brains compared to that found in the postnatal, and especially, in the adult CNS is responsible for promoting the outgrowth and guidance of axons as well as supporting neuronal plasticity 110, 111, 112, 113. Moreover, the 6‐*O*‐sulfation in CS is upregulated after an injury to the CNS and this may be due to the creation of a more permissive environment for axonal regeneration 110, 111, 112. A progressive increase in the content of 6‐*O*‐sulfated CS disaccharides is also detected in cartilage as it ages and seems to be crucial for the maturation and maintenance of this tissue 114. In turn, the cartilage zones in which the osteoarthritis changes are localized show a reduced expression of CS/DS sulfotransferases, especially C6ST‐1 115. Interestingly, the mature osteoarthritis cartilage contains CS with an especially high level of 6‐*O*‐sulfation 116, which could functionally compensate for the loss of a GAG that has such a composition in the disease‐affected tissue areas. An increased content of 6‐*O*‐sulfated CS disaccharides and an elevated deposition of CS/DS have also been shown to characterize the tissue remodeling that is associated with fibrosis 117, 118. However, in contrast to the metabolic changes of CS/DS in the tumor microenvironment, in the fibrosis‐affected tissues there is also a proportional elevation of the 4‐*O*‐sulfation in CS/DS so that the normal ratio between the 6‐*O*‐ and 4‐*O*‐sulfated disaccharides is preserved in these GAGs 117, 118. Interestingly, a recent study using an animal model of lung fibrosis showed that substantial alterations in the CS/DS metabolism begin in the inflammatory phase of this process and that they are regulated by TGFβ1, which also plays a crucial role in the tumor progression 117. To summarize, all of the above‐mentioned data indicate that the 6‐*O*‐sulfation in CS/DS can have important functions that influence cell behavior and/or the ECM properties.

## Monosulfated disaccharides provide interaction surfaces for CS and DS

It is commonly accepted that the CS/DS chain sequence determines the biological properties of these GAGs by creating a suitable chemical surface for the molecular interactions. However, studies that address this issue are especially difficult and arduous, and for these reasons, they are very scarce 119, 120, 121. A recent investigation was focused on the interactions between the chondroitinase B‐resistant CS/DS oligosaccharides and PTN 28. It showed that unbound oligosaccharides were almost exclusively composed of monosulfated disaccharides (that is 4‐*O*‐ and/or 6‐*O*‐sulfated ones), with a minor participation of unsulfated ones 28. However, those species that strongly bound to PTN had a mixed composition being clusters of disulfated as well as monosulfated disaccharides 28. In addition to their interaction with PTN, the sections of the CS/DS chain that accumulate monosulfated and unsulfated disaccharides may also bind poorly to other molecules such as FGF‐2, PDGF‐BB, fibronectin or collagen type III. This conclusion is based on the recent observation that only the monosulfated and unsulfated disaccharides that are probably unengaged in the intermolecular interactions are accessible to chondroitinase ABC and are liberated by the enzyme from the high‐molecular mass complexes that are composed of CS/DS PG and those growth factors and ECM proteins 122.

Further insight into the role of monosulfated disaccharides in CS binding to the growth factors comes from the pioneering study of Sugiura *et al*. 104, who chemo‐enzymatically synthesized CS species with a defined composition and examined their affinity for PTN and MDK. This study allowed the CS structure‐dependent hierarchy in this GAG affinity for both growth factors to be determined. CS species that contained monosulfated disaccharides of the same type were weaker binding partners for PTN and MDK than CS that was a copolymer of the 4‐*O*‐ and 6‐*O*‐sulfated disaccharides 104. In turn, all CSs that contained only monosulfated disaccharides had a significantly lower affinity for the examined growth factors than the GAG that had both monosulfated (4‐*O*‐sulfated) and disulfated (2,6‐*O*‐disulfated) disaccharides 104. Notably, the latter synthetic CS belonged to the strongest binding partners for both growth factors, and had a significantly higher affinity for them than the majority of the CS species that were composed exclusively of disulfated or even trisulfated disaccharides 104. These results suggest that the monosulfated disaccharides that are assembled together with disulfated ones into the binding sequences in CS/DS can actively contribute to the intermolecular interactions of these GAGs. Further evidence to support this suggestion comes from a recent study of Miyachi *et al*. 123, who reported that the CS tetrasaccharides that contained both disulfated and monosulfated disaccharide were recognized by FGF‐2 as strongly as the CS tertasaccharides that contained only disulfated disaccharides. Moreover, the interaction with FGF‐2 requires the appropriate sequence of CS tetrasaccharides with such a mixed composition – the location of the monosulfated disaccharide at the reducing end of such an oligo leads to the binding being voided 123. On the other hand, both this study 123 and others 104, 124, 125 revealed a low affinity or a low binding capacity of the 4‐*O*‐sulfated disaccharide and the semi‐synthetic CSs that contained this disaccharide for some ligands. This property may result from the high conformational rigidity of the 4‐*O*‐sulfated CS disaccharide. The CS backbone consists of alternating GlcA and GalNAc residues that both assume only the ^4^C_1_ conformation. However, some flexibility of this backbone that improves fitting the CS chain to a bound molecule can be regulated by rotation around the glycosidic linkages, which is defined by the pair of dihedral angles ψ and φ. To assess the conformational flexibility of various CS disaccharides, free energies have recently been determined to be a function of the disaccharide backbone geometry using molecular dynamics simulations with all‐atom explicit‐solvent force field technology 126. Using this method, two sequential variants of the unsulfated CS disaccharide (i.e. GlcA‐GalNAc and GalNAc‐GlcA) were found to display the greatest backbone flexibility compared to the corresponding sulfated analogues 126. By contrast, all of the disulfated CS disaccharides revealed the greatest conformational rigidity 126. Moreover, it has been reported that in contrast to the 6‐*O*‐sulfation, the addition of a sulfate moiety to the position at the 4 C of the GalNAc residue in the monosulfated disaccharide significantly reduced the rotation of the disaccharide backbone compared to the geometry of the unsulfated disaccharide 126. These restrictions affected the flexibility of the backbone at the reducing side, but especially at the non‐reducing side of the 4‐*O*‐sulfated GalNAc residue 126. Thus, because the monosulfated disaccharides are predominant elements in the CS composition, any marked alterations in the proportion between the 6‐*O*‐sulfated and 4‐*O*‐sulfated units can significantly modulate the binding potential of this GAG through an influence on the conformational flexibility of the backbone of its chain and/or the proper positioning of the disulfated disaccharides. Interestingly, such a remodeling of the sulfation pattern of the tissue CS/DS is observed during both aging 127 and various diseases 128, 129, 130 and it may be of biological importance. It is conceivable that changes in the ratio of the 6‐*O*‐ to 4‐*O*‐sulfation in CS can even direct this GAG to fulfill special biological functions by strictly determining its binding partner profile. Alternatively, although CS variants that differ in respect to the 6‐*O*‐ to 4‐*O*‐sulfation ratio are able to bind to the same ligand, these interactions could induce slightly different conformational alterations in the ligand molecules. This suggestion is supported by the observation that the interactions of different CS/DS hexamers with interleukin (IL)‐8 caused slightly distinct alterations in the nuclear magnetic resonance spectra of the cytokine 131. It is tempting to speculate that such subtle alterations in the conformation of the ligand molecules resulting from their binding to structurally different CS/DS could, however, markedly modulate the biological properties of the ligand. On the other hand, considering the influence of the position of a sulfate group in the CS backbone on this GAG‐binding properties, the accessibility of these groups for binding should also be taken into account. It is commonly accepted that in a hydrated environment, the CS chains assume a helical conformation with the 6‐*O*‐sulfated groups that are localized peripherally and the 4‐*O*‐sulfated ones that lie near the midline of the polymer 125, 132, 133. Thus, the former groups are more accessible to ligands compared to the latter ones. To summarize the above‐mentioned data, it can be concluded that an increased level of 6‐*O*‐sulfation can induce a high binding potential for CS. This suggestion is also confirmed by the results of a recent study that examined the interactions of semi‐synthetic CSs with several growth and neurotrophic factors 125. Interestingly, these synthetic GAGs that shared some of the features of the sulfation pattern such as a high content of 6‐*O*‐ and unsulfated disaccharides with the CS from the cancer niche were also the strongest binding partners for the majority of growth and neurotrophic factors that were tested 125. On the other hand, the recognition of a CS chain sequence is the only proper way to better understand the biological properties of this GAG. However, only the composition of C‐6‐S, which accumulates in the tumor niche, is known at this time. This limitation also refers to almost all of the investigations that test the biological functions of C‐6‐S. Thus, the results of these investigations can only be considered to be an approximation of the biological effects that C‐6‐S exerts in the tumor niche.

## The role of C‐6‐S in the tumor niche

### C‐6‐S can influence tumor‐associated inflammation

**Figure 2:**
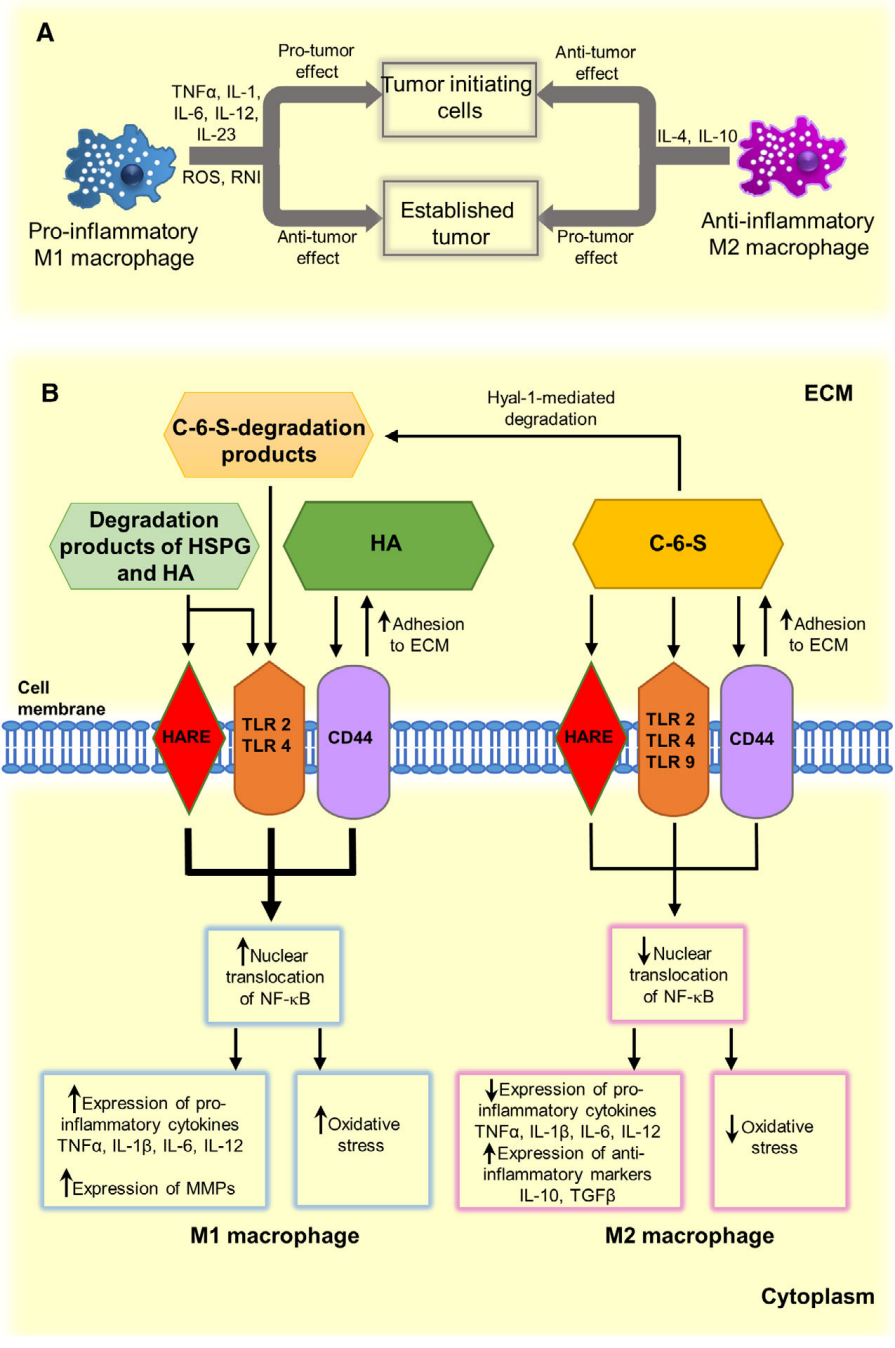
The role of macrophages in the development and progression of cancer (A) and possible regulatory effects of chondroitin‐6‐sulfate on functions of these cells in the tumor microenvironment (B). C‐6‐S, chondroitin‐6‐sulfate; ECM, extracellular matrix; HA, hyaluronan; HARE, HA receptor for endocytosis; HSPG, heparan sulfate proteoglycan; Hyal, hyaluronidase; IL, interleukin; MMP, matrix metalloproteinase; NF‐κB, nuclear factor‐κB; RNI, reactive nitrogen intermediates; ROS, reactive oxygen species; TGFβ, transforming growth factor β; TLR, Toll‐like receptor; TNFα, tumor necrosis factor α.

It is commonly accepted that the development of the majority of, if not all, tumors is tightly linked to a chronic low‐grade inflammation, which is characterized by tissue infiltration by innate and adaptive immune cells (for review: 134, 135). Some of these cells, that is macrophages and neutrophils, produce significant amounts of the reactive oxygen species and reactive nitrogen intermediates that trigger severe alterations in the cell genome, including DNA damage and disturbances in its repair, and an increased rate of genetic mutations and genomic instability, which lead to the initiation of malignant transformations. Another mechanism that is involved not only in the inflammation‐dependent initiation of a tumor, but especially in its progression is an increase in the local concentration of various growth factors, cytokines and chemokines, which are synthesized by both the tumor cells and the tumor‐associated non‐transformed cells, especially macrophages. The correct spatiotemporal expression profile of these mediators in the tumor microenvironment can be crucial for tumorigenesis because the final biological effect of their action strongly depends on the developmental context of a tumor (Fig. 2A). Typical proinflammatory cytokines such as tumor necrosis factor α, IL‐1, IL‐6, IL‐12 or IL‐23, which are primarily synthesized by the type M1 macrophages, manifest protumor activity when they are expressed in the initial stages of the development of a tumor 134. However, the same mediators also have a tumoricidal effect toward established tumors 134. In turn, the anti‐inflammatory cytokines (IL‐4 or IL‐10) that are secreted by the macrophages polarized to the M2 phenotype have an antitumor activity in the initial stages of the tumor development and tumor‐promoting properties toward the established neoplasm 134. The biological effects of the cytokines and growth factors result from the impact of these molecules on the expression of the downstream effectors such as other cytokines and growth factors as well as angiogenic factors, adhesion molecules, ECM components or various enzymes that are responsible for ECM processing. This impact occurs via the modulation of the activity of several transcription factors (mainly nuclear factor (NF)‐κB, activator protein 1 and/or signal transducer and activator of transcription 3), which affects the proliferation, survival, adhesion and migration of malignant cells as well as the vascularization of a tumor 135.

Chondroitin sulfate is known for its immunomodulatory activity. Several *in vitr*o and *in vivo* studies have shown that CS can reduce oxidative stress and/or diminish the biosynthesis of various proinflammatory molecules in proinflammatory‐stimulated cells 136, 137, 138, 139, 140, 141, 142. For this reason, CS was introduced as a dietary supplement for the treatment of patients suffering from osteoarthritis 143. However, the CS‐mediated influence on inflammation may be cell‐specific and, more importantly, it may depend on the GAG structure, especially on the sulfation pattern. Such a suggestion results from recent reports that have examined the influence of CS that differ in respect to their predominant sulfation model on the severity of experimental autoimmune encephalomyelitis. Administration of C‐4‐S in an animal model of experimental autoimmune encephalomyelitis exacerbated the inflammation 144. By contrast, experiments with a knockout and overexpression of C6ST‐1 revealed that 6‐*O*‐sulfated CS can alleviate a clinical manifestation of the disease 145. It has also been shown that C‐6‐S inhibited the *in vitro* secretion of IL‐6 in macrophages, which were proinflammatorily stimulated with CpG via Toll‐like receptor (TLR) 9, more effectively than C‐4‐S 133. Notably, the impact of CS on macrophage activity can be a crucial issue in the development and progression of a tumor as these cells are responsible for creating and maintaining the protumor–antitumor balance. It has been reported that structurally diverse CS preparations significantly reduced the liberation of several proinflammatory molecules from macrophages that had been stimulated with lipopolysaccharide (LPS) 146. However, among those preparations, C‐6‐S inhibited the broadest spectrum of inflammatory mediators 146. Thus, C‐6‐S, which gradually accumulates in the tumor niche, can affect the secretory profile of the resident macrophages there (Fig. 2B), thereby fixing the M2 polarization of these cells 147 and supporting an established tumor 134 (Fig. 2). However, the CS‐mediated impact on specific inflammatory conditions in the tumor microenvironment may be more complex. It has been shown that the oligosaccharides that were generated from C‐6‐S by bovine Hyal strongly stimulated *in vitro* human monocytes to release proinflammatory cytokine IL‐12 148. Importantly, Hyals are among the ECM‐processing enzymes that can be upregulated in the tumor niche 95. Thus, the balance between the C‐6‐S deposition and the C‐6‐S degradation and clearance of its degradation products in the tumor niche rather than just the accumulation of this GAG could, in fact, determine its final effect on tumor‐associated inflammation (Fig. 2B).

### C‐6‐S‐modulated receptor function can affect NF‐κB signaling and cell behavior

The mechanism(s) by which CS attenuates the inflammatory response in cells is poorly known. However, several *in vitro* and *in vivo* studies have reported that in various cells that were simultaneously exposed to inflammatory stimuli and CS, the translocation of NF‐κB from the cytosol to the nucleus was markedly reduced compared to that observed in the only proinflammatorily activated cells 137, 138, 139, 140, 141, 142, 146, 147. Moreover, it has been reported that CS with a high level of 6‐*O*‐sulfation can inhibit the NF‐κB activation in bone marrow macrophages more effectively than the GAG variant with a high level of 4‐*O*‐sulfation 146, 147. In addition to native CS, unsaturated 6‐*O*‐sulfated CS disaccharides are also able to exert this inhibitory effect on NF‐κB 149. However, the intracellular signaling that implicates NF‐κB as a downstream effector not only plays a crucial role in triggering an inflammation but also in the development and progression of a tumor 135. Unfortunately, almost all of the published reports have addressed the CS‐dependent impact on NF‐κB activation on non‐malignant cells. However, it has recently been reported that CS chains of serglycin can unexpectedly trigger the NF‐κB activation in non‐small cell lung cancer (NSCLC) cells, thereby promoting their invasiveness 150. Interestingly, serglycin, which is overexpressed in some tumors such as breast cancer, NSCLC or multiple myeloma and secreted by them 150, 151, 152, 153, is modified by C‐4‐S chains 151, 152.

The CS‐dependent influence on the NF‐κB signaling pathway is most probably transduced via this GAG binding to the cellular receptors. Among the receptors that simultaneously trigger an NF‐κB cascade and that can interact with GAGs there are TLRs, the HA receptor for endocytosis (HARE) and CD44. TLRs, which represent the major class of pattern‐recognition receptors, not only participate in the induction and regulation of inflammatory and tissue repair responses to injury but also play a dual role in the development and progression of a tumor 154. On one hand, TLR activation is responsible for clearly antitumor effects such as the recruitment of tumor cytotoxic cells (natural killer cells and cytotoxic T cells), the conversion of the macrophage phenotype from M2 to M1 as well as the interruption of the tolerance to tumor‐self antigens 154, 155. On the other hand, various *in vitro* and *in vivo* studies have shown that the stimulation of TLRs (mainly TLR2 and TLR4, which are localized on both tumor cells and tumor‐associated host cells) leads to an increase in the survival, proliferation and metastatic potential of tumor cells 156, 157, 158. In contrast to HA or heparan sulfate, CS is not a typical ligand for TLR2 and TLR4 159, 160. However, there is some evidence that CS can interact with and affect TLR function. For instance, the anti‐inflammatory effect of C‐6‐S (or C‐4‐S) on chondrocytes that had been stimulated with LPS via TLR4 was lessened when these cells were treated with anti‐TLR4–M2 complex antibody prior to the administration of the GAG 141. Moreover, chondrocytes that were first treated with C‐6‐S (or C‐4‐S) and then with LPS displayed a significant reduction in the inflammatory response compared to the cells that had only been stimulated with LPS 141. Additionally, both native CS (especially C‐6‐S) and the CS‐degradation products can effectively inhibit the biological effects that are induced by TLR1/2 and ‐9 133. Thus, it is possible that C‐6‐S could directly interact with TLRs in the tumor microenvironment, thereby interfering in the ligand–receptor binding and/or attenuating the signal transduction and downstream signaling. A similar mechanism may be associated with the effect of C‐6‐S on HARE and CD44, which are also upstream elements in the NF‐κB cascade. HARE, which is mainly located on the surface of the liver sinusoidal cells and macrophages, is a primary scavenger receptor for the systemic clearance of modified low‐density lipoproteins and apoptotic cells as well as GAG degradation products 161, 162. It was shown 162 that the binding of C‐6‐S to HARE inhibited the interaction of this receptor with HA and blocked the NF‐κB activation in macrophages. In turn, CD44 is a major HA‐binding cell surface receptor that is ubiquitously expressed on cells such as leukocytes, fibroblasts and cancer cells 163. Moreover, this receptor is involved in promoting the survival and invasiveness of cancer cells 163. In macrophages that had been exposed to IL‐4, the so‐called standard variant of CD44 (sCD44) is modified by the CS chain that interacts with this receptor and inhibits its binding to HA 164. It is hypothesized that CS binding may lead to conformational alterations in the receptor, which reduce its affinity to HA 164. Interestingly, the specific immunoreactivity of the CS attached to sCD44 and an increased expression of C6ST‐1 in cells that had been exposed to IL‐4 suggest that these inhibitory properties of the GAG are associated with an enhanced level of its 6‐*O*‐sulfation 164. This possibility is further supported by the observation that C‐6‐S is a good binding partner for sCD44 165. Thus, C‐6‐S could play a role as a specific modulator of the activity of cell‐surface receptors through the interaction with them and the induction of conformational alterations in their molecules, which could affect downstream signaling. Interestingly, the stimulation of FGF receptor‐1 with a combination of FGF‐2 and heparin triggers a different tyrosine phosphorylation in the receptor molecule compared with the one that is induced by the growth factor alone 166.

**Figure 3:**
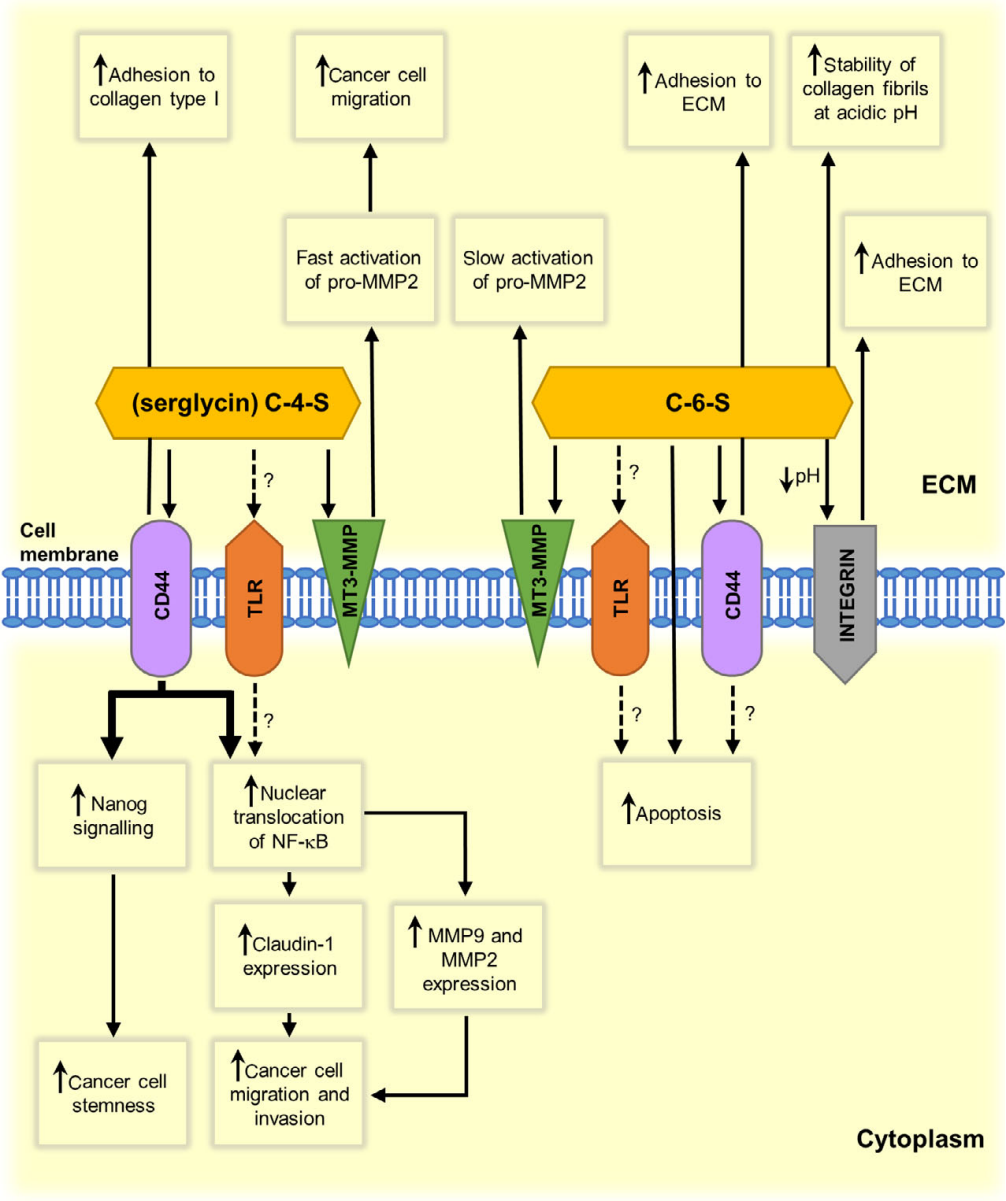
Comparison of chondroitin‐6‐sulfate‐ and chondroitin‐4‐sulfate‐mediated effects on cancer cell behavior and/or extracellular matrix properties in the cancer niche. Dashed lines indicate unproven but possible mechanisms. CS, chondroitin sulfate; ECM, extracellular matrix; MMP, matrix metalloproteinase; MT‐MMP, membrane‐type matrix metalloproteinase; NF‐κB, nuclear factor‐κB; TLR, Toll‐like receptor.

Evidence supporting the suggestion that the 6‐*O*‐sulfation of CS can strongly influence the behavior of a tumor cells (Fig. 3) has come from the observation that xyloside‐primed CS/DS that was produced by a breast carcinoma cell line demonstrated a strong cytotoxic activity toward both normal and tumor cells 167, 168. This effect, which involved the induction of apoptosis, was neutralized by cancer‐cell‐synthesized heparan sulfate 167. Interestingly, CS/DS with these cytotoxic properties had a very high proportion of the 6‐*O*‐sulfated disaccharides in relation to the 4‐*O*‐sulfated ones (82–63% *versus* 15–30%) compared to the cytotoxically inactive GAG from normal breast fibroblasts, which contained ~60% of the 4‐*O*‐sulfated disaccharides and ~34% of the 6‐*O*‐sulfated ones 167, 168. Moreover, the cytotoxic CS/DS also differed from the GAG that was produced by normal cells in respect to a significantly lower content of the IdoA residues (~10% *versus* ~40%, respectively) and to the occurrence of 4,6‐*O*‐disulfated disaccharides (E units) 167, 168. However, despite their known biological potential, the presence of E units cannot be the only structural determinant that is responsible for the cytotoxic effect of the xyloside‐primed CS/DS because another CS/DS that was used in the research and that had a similar level of these disaccharides did not demonstrate any cytotoxic activity 167. Thus, the sequential context in which E units are presented such, as the 6‐*O*‐ or 4‐*O*‐sulfation of adjacent disaccharides, may be crucial for the biological properties of CS/DS. This hypothesis is further supported by the observation that the CS chains of cancer cell‐derived serglycin, which are composed of a small number of 4,6‐disulfated disaccharides in addition to the predominant 4‐*O*‐sulfated ones (from 87 to 93% of the total disaccharides *versus* maximally 10% of 6‐*O*‐sulfated and 5% of unsulfated ones) 151, 152, strongly promote the anchorage‐independent growth of these cells 151. Serglycin, which is secreted by NSCLC, mediates this effect via the interaction of its CS with CD44 and the induction of Nanog signaling, thereby stimulating cancer cells’ stemness and enhancing their chemoresistance 150 (Fig. 3). The secretion of serglycin is also positively correlated with the aggressiveness of breast cancer cells 151. Moreover, this PG stimulates the *in vivo* progression of multiple myeloma most probably through the influence of its CS on the delivery of proangiogenic factors such as HGF 153.

### C‐6‐S as a regulator of cytokine/chemokine activity

The immunomodulatory properties of CS also result from the direct binding of this GAG to various cytokines, followed by a modulation of the cytokine activity. IL‐10 is one of the cytokines that interact with CS. This cytokine, which is a key regulator of the innate and adaptive immune systems, controls the inflammatory response by downregulation of the proinflammatory cytokines and cell‐surface molecules involved in antigen recognition 169. Interestingly, bone marrow macrophages that had been exposed to C‐6‐S had an overexpression of IL‐10 147. However, C‐6‐S not only influences the level of IL‐10 synthesis but also affects other aspects of cytokine biology. An examination of the GAG interactions with IL‐10 using nuclear magnetic resonance spectroscopy showed that the 6‐*O*‐sulfated CS disaccharide demonstrated the lowest affinity for the cytokine among all of the CS and heparin disaccharides 170. These data suggest that ECM that is abundant in C‐6‐S could ensure the high bio‐availability of IL‐10 for cells due to a reduced level of its sequestration. Furthermore, compared to other sulfated GAGs, C‐6‐S interferes with the biological effects of IL‐10 the least 171. Thus, all of these findings suggest that C‐6‐S can be a significant promoter of the anti‐inflammatory activity of IL‐10 in the tumor microenvironment, thereby preventing the initiation of a tumor or supporting the progression of an established tumor. GAGs are also crucial regulators of the chemoattractive cytokines, called chemokines. The interactions of chemokines with the ECM‐localized GAGs protect these cytokines from proteolysis and enable them to form the established gradient that is responsible for the directional migration of leukocytes, which supports the recruitment of these cells to areas with tissue injury 172. Moreover, GAG chains create a surface for chemokine oligomerization or for the stabilization of the existing chemokine oligomers, which can affect the activation of receptors for these cytokines 173. Recently it was shown that intracellular CS contributes to the mechanism that regulates intracellular trafficking and the secretion of monocyte chemoattractant protein 1 in breast cancer cells 174. Despite numerous studies that examine the role of GAGs in chemokine biology, investigations that focus on the impact of C‐6‐S on these mediators are scarce. However, it has been shown that C‐6‐S hexasaccharide is a better binding partner for IL‐8 than these oligos that are derived from C‐4‐S, DS or HA 131. Moreover, CS can also affect the activity of this chemokine as a result of the observation that this GAG strongly amplifies the IL‐8‐mediated generation of reactive oxygen species in neutrophils 175. Thus, all of these findings suggest that the progressive accumulation of C‐6‐S in the tumor microenvironment together with an enhanced local secretion of IL‐8 by cancer‐associated cells creates the conditions that support the recruitment of neutrophils and their activation. A high neutrophil infiltration of tumor tissues strongly correlates with a poor outcome in patients since these cells are the source of the ECM‐remodeling enzymes and proangiogenic factors such as IL‐8 176. Thus, C‐6‐S binding to IL‐8 can protect a chemokine from proteolysis, thereby also stimulating its proangiogenic effect.

### The effect of C‐6‐S on the invasive properties of cancer cells

Metastasis is a crucial process in the progression of cancer that is responsible for cancer mortality to the greatest extent 135. This process includes four major steps: (a) the mesenchymal transformation of the primary epithelial cancer cells (epithelial–mesenchymal transition); (b) the migration of tumor cells across tissues into the blood or lymphatic vessels (intravasation); (c) the travel of cancer cells throughout the circulation, and finally, (d) the extravasation as well as colonization of new tissues 135. For all of these events features of cancer cells such as their ability to adhere to various ECM and cell surface molecules (collagen, fibronectin, selectins, CD44, ICAM and vascular cell adhesion molecule) as well as increased mobility throughout the actively remodeled ECM are of special importance. Growing evidence suggests that CS that is localized in the tumor ECM and on the tumor cell surface can play a dual role in cancer metastasis. Recently it was shown in an animal model of breast cancer that the enzymatic degradation of CS/DS within a primary orthotopic tumor did not affect the growth of the primary tumor; instead it induced lung metastasis 177. On the other hand, a high CS expression in malignant cells but not in the tumor stroma strongly correlates with a brief recurrence‐free survival and an overall survival in breast cancer patients 63. The significant influence of the CS that is present in the cancer cell glycocalyx on various aspects of the invasiveness of cancer cells has been shown in several *in vitro* investigations. The so‐called oncofetal type of CS that is attached to several osteosarcoma cell‐surface PGs seems to stimulate the migration and invasion of these cells as it was observed that the functional inhibition of this GAG via its binding to a *Plasmodium falciparum*‐derived VAR2CSA protein significantly reduced both integrin signaling and cancer cell motility 178. Furthermore, the CS, which is attached to CSPG4 on the melanoma cell surface, promotes the α4β1‐dependent adhesion of these cells to fibronectin and the associated signaling via an interaction with the α4 integrin subunit 179. In turn, the CS moiety of CD44 mediates the migration of melanoma cells on type IV collagen but not the adhesion to this substratum that engages α2β1 integrin 180. Moreover, the interaction between the CD44 from the surface of multiple myeloma cells and the C‐4‐S chains of serglycin that they secrete is responsible for the adhesion of these cancer cells to collagen type I and to bone marrow stromal cells, which then enables bone marrow colonization 153 (Fig. 3). Additionally, the adhesion of multiple myeloma cells to collagen type I via an interaction that involves serglycin CS stimulates these cells to synthesize and secrete matrix metalloproteinase (MMP)‐9 and MMP‐2, which promotes the invasiveness of cancer cells via ECM remodeling 181 (Fig. 3). The CS moiety of serglycin also potentiates the invasiveness of breast cancer and NSCLC 150, 151. The mechanism that underlies this effect in the NSCLC cells implicates the activation of the CD44–NF‐κB–claudin‐1 axis 150 (Fig. 3). CD44, which is modified by CS, via this GAG binds to fibronectin, thereby supporting cell adhesion 182. In addition, the CD44‐spliced variant that contains the sequence encoded by the v10 exon can interact with the CS that is localized extracellularly or on the cell surface using the B[X7]B motif (where B is a basic amino acid and X is an any amino acid) that is present within this sequence 183. Since such a binding can influence cell–ECM and cell–cell adhesion, the expression of the v10 CD44 isoform on cancer cells can enhance their metastatic potential 183. Similarly, as in the case of its impact on the development of a tumor, the influence of CS on metastasis also seems to be determined by the sulfation pattern of this GAG. Notably, several studies have demonstrated a complex effect of the E disaccharides on the dissemination of cancer. CS‐E (i.e. CS that contains an increased level of the 4,6‐*O*‐disulfated disaccharides) has been shown to substantially reduce the invasiveness of breast cancer cells *in vitro* inhibiting Wnt/β‐catenin signaling, which results in the downregulation of the prometastatic *Col1*α*1* gene 184. Moreover, CS‐E can impede the ECM processing, which favors cancer cell mobility, by both enhancing retention of tissue inhibitor of metalloproteinases (TIMP)‐3 in the extracellular space and by stimulating its inhibitory effect toward adamalysin‐like metalloproteinase with thrombospondin motifs‐5 185. By contrast, the metastatic potential of Lewis lung carcinoma cells or murine osteosarcoma cells positively correlates with the proportion of the E units in the CS that are localized on the cell surface 87, 88, 186. Likewise, the overexpression of CS‐E in ovarian cancer cells correlates with a poor prognosis 187 most probably due to an increase in the cell–ECM and cell–cell adhesiveness 188 that can facilitate the tissue colonization. It seems that the mechanisms that underlie the prometastatic activity of CS‐E may involve effects on the cell motility stimulators such as VEGF and HGF 189, 190, the promotion of circulating cancer cell survival and extravasation via the interaction with P selectin 191, support of tissue colonization by binding to local receptors such as RAGE 46, and the stimulation of activation and/or activity of enzymes responsible for ECM degradation such as MMP‐7 192, which is implicated in the disruption of cell‐ECM contacts 193. Moreover, the CS‐E degradation products strongly stimulate CD44 proteolysis, thereby increasing the CD44‐dependent migration of cancer cells 194. On the other hand, the fact that CS‐E can demonstrate both a prometastatic and an antimetastatic activity indicates the complexity of the intermolecular connection network in which this GAG participates. However, it cannot be excluded that the final biological effects that are promoted by the E units can also be controlled by molecular context (i.e. the CS‐E sequence) rather than being solely dependent on the presence of these disaccharides. In contrast to CS‐E, the role of CS with a high level of 6‐*O*‐sulfation in the dissemination of cancer is still poorly known. In contrast to the expression of C4ST‐1, the *in vitro* expression of C6ST‐1 is not correlated with the aggressiveness of breast cancer cells 195. C‐6‐S also did not influence the invasive phenotype of mouse breast cancer cells that had been grown in 3D cultures 184. Moreover, C‐6‐S had a low affinity for P selectin 191. Taken together, these results suggest that this GAG has at least a neutral effect on the metastasis. On the other hand, C‐6‐S avidly binds to sCD44 and this interaction can promote both cell adhesion and cell migration 165. Thus, a gradual accumulation of C‐6‐S in the tumor stroma can not only facilitate the settlement of the tumor environment by cells that display a high level of sCD44 expression such as leukocytes and fibroblasts 163, but also support the invasive potential of cancer cells. However, the impact of C‐6‐S on the invasiveness of cancer is more complex. It was shown that C‐6‐S significantly decreased both the migration and the invasion of melanoma cells that were co‐cultured with normal fibroblasts 196 (Fig. 3). The underlying mechanism may involve an increase in the integrin‐mediated adhesion of cancer cells to the ECM as a consequence of a C‐6‐S‐dependent acidification of the pericellular space 196. Furthermore, the migration of cancer cells across tissue involves the focal proteolysis of the ECM components at the invasive edge of the migrating cells, which is mediated by cell surface‐associated enzymes such as MMP‐2. The activation of pro‐MMP‐2 by membrane‐type 3 MMP is facilitated by CS and this effect depends on the GAG sulfation 197. C‐4‐S is markedly more effective in this process than C‐6‐S 197. Therefore, by this mechanism, the C‐6‐S that is deposited in the cancer microenvironment could retard both the ECM remodeling and the mobility of cancer cells compared to the C‐4‐S action (Fig. 3). In addition, C‐6‐S does not stimulate MMP‐7 activity 192. However, this GAG also does not improve the extracellular retention of TIMP‐3 185. On the other hand, C‐6‐S inhibits the proteolytic activity of cathepsin S less effectively than C‐4‐S 198. This enzyme is engaged in a broad spectrum of events that support the invasiveness of cancer such as angiogenesis or the disruption of cell–cell and cell–ECM contacts 199. However, C‐6‐S may also affect the metastasis by influencing the collagen network in the tumor niche. It has been reported that the biomechanical properties of ECM, in particular the enhanced stiffness of the collagen network due to the excessive deposition of this protein as well as its increased cross‐linking by lysyl oxidase, strongly affect the invasiveness of many types of cancer 3, 200. In addition, the presence of long collagen fibers in the tumor stroma is correlated with a poor survival outcome in multiple types of cancer 201. Though the detailed mechanism that underlies the formation of such fibers in the tumor stroma is unknown, it is well evidenced that collagen fibrogenesis is regulated by CS/DS PG 202. Moreover, it has been reported using high‐resolution scanning microscopy that the morphology of the collagen network is substantially affected by the sulfation of CS 203. In contrast to C‐4‐S, C‐6‐S was entirely bound to the fibril surface and influenced the fibril parameters such as their diameter and spatial layout 203. Recently, it has been shown that C‐6‐S binds to and preserves collagen fibrils from acidic pH‐dependent denaturation/solubilization 204 (Fig. 3). Interestingly, C‐6‐S can be more effective in this protective effect than DS 204. Moreover, interactions between C‐6‐S and collagen lead to an increased resistance of collagen fibrils to cathepsin K‐mediated degradation 204. It is possible that similar C‐6‐S‐dependent effects occur on the collagen in the acidic tumor environment and are responsible for the remodeling of the collagen network that can affect cancer cell motility 196, 200, 201.

In the light of the above‐mediated data, it can be concluded that the C‐6‐S that is accumulated in the tumor niche can indirectly influence the invasiveness of cancer cells by supporting the adhesion of various host cells such as fibroblasts or leukocytes. These cells are significant sources of growth factors and ECM‐degrading enzymes, which stimulate the migration of cancer cells. Furthermore, C‐6‐S can modulate the ECM architecture of the tumor niche and its processing, thus directly affecting the dissemination of cancer cells.

## Future perspectives

The presented data suggest that C‐6‐S plays a dual role in the microenvironment of a tumor. However, in order to take advantage of these data in anticancer therapy, a deep insight into the C‐6‐S sequence as well as its relation to the GAG function is required 89. Although there are new promising techniques for sulfated GAG sequencing 205, only laboratory practice can verify their effectiveness. Nevertheless, in light of the current knowledge about the contribution of C‐6‐S in the development, progression and spread of cancer, several therapeutic strategies that target this GAG can be proposed. First and foremost, due to its anti‐inflammatory properties, C‐6‐S can be applied as a slow‐acting anti‐inflammatory drug for the prevention of cancer. Moreover, taking into account its ability to interact with CD44 165, C‐6‐S can be a target for therapy to diminish the infiltration of the cancer niche by host cells such as fibroblasts, which are hypothesized to be the primary mediators of drug resistance in cancer cells 206. On the other hand, due to its negative electrical charge and binding to several cell‐surface receptors such as HARE 162 or the above‐mentioned CD44 165, C‐6‐S or its chain fragments could also be applied as drug delivery carriers in order to enhance the specificity and efficiency of anticancer therapy 207.

## Conflicts of interest

The authors declare no conflict of interest.

## Author contributions

AP conceptualized and wrote part of the text (section 1, 2 and partly 3) as well as preparing Table 1. GW prepared all figures and edited the text. KO read the text critically. EMK conceptualized and wrote the remaining text as well as coordinating the final form of the manuscript.
